# Different Views about Work-Hour Limitations in Medicine: A Qualitative Content Analysis of Surgeons', Lawyers', and Pilots' Positive and Negative Arguments

**DOI:** 10.1371/journal.pone.0113578

**Published:** 2014-11-24

**Authors:** Adrian P. Businger, Reto M. Kaderli

**Affiliations:** 1 Military Medical Service, Swiss Armed Forces, Ittigen-Bern, Switzerland; 2 Private University in the Principality of Liechtenstein, Triesen, Liechtenstein; 3 Department of Visceral Surgery and Medicine, Bern University Hospital, University of Bern, Bern, Switzerland; Cardiff University, United Kingdom

## Abstract

**Background:**

Whereas work-hour regulations have been taken for granted since 1940 in other occupational settings, such as commercial aviation, they have been implemented only recently in medical professions, where they lead to a lively debate. The aim of the present study was to evaluate arguments in favour of and against work-hour limitations in medicine given by Swiss surgeons, lawyers, and pilots.

**Methods:**

An electronic questionnaire survey with four free-response items addressing the question of what arguments speak in favour of or against work-hour limitations in general and in medicine was sent to a random sample of board-certified surgeons, lawyers in labour law, and pilots from SWISS International Airlines Ltd.

**Results:**

In all, 279/497 (56%) of the respondents answered the survey: 67/117 surgeons, 92/226 lawyers, and 120/154 pilots. Support for work-hour limitations in general and in medicine was present and higher among lawyers and pilots than it was in surgeons (p<0.001). The latter agreed more with work-hour limitations in general than in medicine (p<0.001). The most often cited arguments in favour of work-hour limitations were “quality and patient safety,” “health and fitness,” and “leisure and work-family balance,” whereas the lack of “flexibility” was the most important argument against. Surgeons expected more often that their “education” and the “quality of their work” would be threatened (p<0.001).

**Conclusions:**

Work-hour limitations should be supported in medicine also, but a way must be found to reduce problems resulting from discontinuity in patient care and to minimise the work in medicine, which has no education value.

## Background

The circumstances that led to the tragic death of Libby Zion in a New York teaching hospital in 1984 opened a debate about patient safety in medicine [1]. It has been recognised that relatively moderate levels of fatigue impair performance in a way quite similar to that of alcohol intoxication [2], which could be a safety risk for patients. Extended work shifts were found to be linked with more serious medical errors and impaired performance in simulated surgical tasks [3]–[5]. As a result of a subsequent broad political discussion, regulations of doctors' work hours in Western countries followed. What had been taken for granted since 1940 in other occupational settings, such as commercial aviation, namely strict work-hour regulations and rest periods [6], was completely new for medical professions. Regulations to limit pilot flight time and rest times were strict and above all initiated to eliminate midair pilot fatigue caused by long working hours, which could put the plane and its passengers at great risk [7]. Regarding medical professions, the number of work hours was limited differently in diverse industrialised countries, without any evidence regarding an ideal. Whereas the US Accreditation Council for Graduate Medical Education implemented nationwide duty-hours standards beginning July 1, 2003, with work hours limited to 80 hours per week for junior doctors [8], work-hour limitations for all residency programs in Switzerland were set on January 1, 2005, by the Swiss government [9]. They include a 50-hour weekly limit with a maximum overtime of 2 hours per day and 140 hours per year, respectively, and at least 11 hours of rest between duty periods. Overtime per day may exceed 2 hours during work-free business days or in emergency cases. Daily rest time may be reduced to 9 hours several times per week, as long as the average resting time over a two-week period amounts to 12 hours daily.

The implementation of work-hour limitations developed into a lively debate with, on the one side, the positive effects of ameliorating residents' quality of life and giving them more time to maintain a social network [10],[11] and on the other side, the threat to the Halsted paradigm of unconditional dedication to training with negative impacts on surgical education owing to reduced caseloads [11]–[13]. However, because work hours are frequently underreported by residents [14], they did not decrease uniformly with the implementation of work-hour limitations. Furthermore, the effect on patients' safety is contradictory as well, with little evidence of improvement from restricting residents' work hours [15]–[18].

With regard to ongoing policy debates about work-hour limitations in medicine, the aim of this study was to evaluate arguments given by Swiss surgeons in favour of and against work-hour limitations in medicine. For comparison, the same questions were posed to pilots—with their decades of strict work-hour regulations—and to labour law lawyers, so that they could consider the issue from their model guidelines and standards in daily business and experience in legal matters, respectively. The current argumentary in studies is solely lead by physicians.

## Methods

We used an electronic questionnaire survey with four free-response items addressing the question of what arguments speak in favour of or against work-hour limitations for general employees on the one hand to get arguments independent of a certain profession and for physicians working in hospitals on the other hand. The free-response items read as follows: “In your opinion, what arguments are in favour of or against work-hour limitations in general?” and “what arguments are in favour of or against work-hour limitations in medicine?”. In addition, participants were asked to grade their agreement with work-hour limitations in general and in medicine (Likert scale of 1 to 5: 1 = completely disagree, 2 = rather disagree, 3 = neither agree nor disagree, 4 = rather agree, 5 = completely agree). We gathered additional information including sociodemographic characteristics (age, sex, marital status), work characteristics (hours worked, work experience, part-time work), and hierarchy. The survey was pre-tested for acceptability and clarity in an interdisciplinary focus group consisting of surgeons, pilots and lawyers, respectively.

An outline of this study was evaluated by the Cantonal Ethical Committee of Graubunden, Switzerland. The ethical committee did not provide a specific waiver for the present study. Completion of the survey comprised written informed consent to participate in the study. The Swiss Surgical Society database and the Swiss Bar Association were freely accessible to everyone during the time of our study. We obtained anonymous data from Heiner Hoffmann from the SWISS International Airlines Ltd database because these data were not freely accessible to us. To ensure the participants' anonymity, data from participant questionnaires were entered into an anonymous database. Participants had the option to provide us their e-mail addresses if they wished discuss the issue further.

### Participants

A random sample of 15% of board-certified surgeons and labour law lawyers registered in the database of the Swiss Surgical Society (n = 783 ordinary members) and with the Swiss Bar Association (n = 1507 members), respectively, were invited to participate in an electronic qualitative survey during 2011/2012 [19],[20]. Additionally, a random sample of 15% of SWISS International Airlines Ltd. Pilots who were members of the Swiss Air Line Pilots Association (SwissALPA) Aeropers, Kloten, Switzerland (n = 1029 members) was contacted.

### Qualitative analysis

An experienced sociologist with particular expert knowledge in qualitative data analysis performed all statistical computations. The arguments in favour of and against work-hour limitations in general and in medicine were evaluated using Mayring's content analysis by first transcribing the respondents' free-response arguments into an Excel file and then defining the level of abstraction to inductively form categories and generate a code manual [21]. In an additional step, the texts were encoded and assigned to the content categories. A formative check of reliability and, finally, a summative check of reliability were performed. Dichotomous variables were analysed by Chi^2^-test.

To explore the impact of age on the arguments in favour of or against work-hour limitations, the participants were split into two groups based on their median age. A split into 5 groups (1–5, 6–10, 11–20, 21–30 and 31–50 years in profession) was performed to analyze the influence of work experience.

## Results

After the first mailing and two reminders, 279/497 (56%) of the respondents answered the survey: 67/117 surgeons, 92/226 lawyers, and 120/154 pilots. Four-fifths were men (229/279, 82.1%). The mean age was 45.3 years (SD 9.6). Most respondents (161/275, 58.5%) were at the highest hierarchical level, 75/275 (27.3%) were at the middle level, and 39/275 (14.2%) held junior posts (4 missing values); most of them (200/277, 72.2%) worked full-time, 71/277 (25.6%) worked part-time (50–99%), and 6/277 (2.2%) worked part-time <50% (2 missing values). On average, respondents had 19.0 years (SD 9.8) of work experience and worked 48.8 hours (SD 16.6) per week (Table 1).

**Table 1:** Demographics of the 279 participants.

Characteristic	Total, N = 279	Surgeons, N = 67	Lawyers, N = 92	Pilots, N = 120	*P*
Age, mean (SD), yrs	45.3±9.6	49.3±8.5	43.8±10	44.2±9.4	<0.001
Male	229 (82.1)	60 (89.6)	53 (57.6)	116 (96.7)	<0.001
Hierarchical status, 4 missing values					0.034
Low (e.g., attending, trainee, first officer)	39 (14.2)	16 (24.2)	6 (6.7)	17 (14.2)	
Medium (e.g., consultant, senior first officer)	75 (27.3)	15 (22.7)	24 (27.0)	36 (30.0)	
High (e.g., chief, partner, captain)	161 (58.5)	35 (53.0)	59 (66.3)	67 (55.8)	
Employment, 2 missing values					<0.001
Full-time	200 (72.2)	62 (92.5)	61 (67.8)	77 (64.2)	
80–99%	51 (18.4)	4 (6.0)	15 (16.7)	32 (26.7)	
50–79%	20 (7.2)	1 (1.5)	9 (10.0)	10 (8.3)	
<50%	6 (2.2)	0 (0)	5 (5.6)	1 (0.8)	
Work hours per week, mean (SD)	48.8±16.6	66.5±9.9	47.2±8.9	35.6±13.9	<0.001
Work experience, mean (SD), yrs	19.0±9.8	21.8±8.8	16.7±10.4	19.4±9.6	0.006
Living with a partner, 4 missing values	223 (81.1)	55 (83.3)	68 (76.4)	100 (83.3)	0.036
Agreement with work-hour limitations ≥4*					
in general, 1 missing value	236 (84.9)	51 (77.3)	69 (75.0)	116 (96.7)	<0.001
in medicine, 8 missing values	222 (81.9)	39 (59.1)	72 (79.2)	111 (97.3)	<0.001

Values in parentheses are percentages.

*Five-point Likert scale: 1 = completely disagree, 2 = rather disagree, 3 = neither agree nor disagree, 4 = rather agree, 5 = completely agree.

### Agreement with work-hour limitations in general and in medicine

The agreement with work-hour limitations in general, ranked on a 5-point Likert scale anchored by 1 = completely disagree and 5 = completely agree, differed among the groups: 3.89±0.99 for surgeons, 3.99±1.03 for lawyers and 4.66±0.65 for pilots; p<0.001. The agreement with work-hour limitations in medicine differed as well: 3.39±1.28 for surgeons, 4.10±1.02 for lawyers, and 4.75±0.57 for pilots; p<0.001. Whereas no difference was found for lawyers and pilots, surgeons agreed more with work-hour limitations in general than with limitations in medicine (p<0.001).

### Arguments in favour of and against work-hour limitations in general and in medicine

The 279 participants gave a total of 1,078 arguments (716 in favour of and 362 against) on the question about work-hour limitations in general and 436 arguments (300 in favour of and 136 against) on the question about work-hour limitations in medicine. The content analysis of the arguments resulted in five categories of arguments in favour of and seven categories against work-hour limitations. The five positive categories were (listed in decreasing frequency distribution of the positive arguments for work-hour limitations in general): “Quality, patient safety” (25.7%), “health, fitness” (25.6%), “leisure, work-family balance” (25.3%), “efficacy” (14.0%) and “satisfaction, pleasure” (8.5%), and the seven negative categories were (listed in decreasing frequency distribution of the negative arguments for work-hour limitations in general): “lack of flexibility” (26.5%), “costs” (19.1%), “education” (16.9%), “quality of work” (14.1%), “organisability” (11.9%), “performance” (3.6%) and “salary” (3.3%). Non-distinctive arguments, such as “work-hour limitations are the beginning of an approach to the work time of officials” or “work-hour limitations are no contemporary form”, were assigned as not codeable. Examples of participants' arguments for and against work-hour limitations are shown in Tables 2 and 3.

**Table 2:** Examples of surgeons', lawyers', and pilots' arguments for work-hour limitations in general and in medicine.

Category of arguments		N (%)	Surgeons	Lawyers	Pilots
Quality, patient safety	In general	184 (25.7)	High quality for patients and physician.	Better quality of performed accomplishments.	Additional safety in general.
	In medicine	122 (40.7)	More safety for patients.	Patient safety.	To prevent mistakes as a result of fatigue.
Health, fitness	In general	183 (25.6)	Improved predictability of mental and physical strain.	Prevention of overstrain.	Less chronic fatigue.
	In medicine	70 (23.3)	Prevention of exhaustion.	No fatigue/burnout.	I don't want to be operated on by an overworked surgeon.
Leisure, work-family balance	In general	181 (25.3)	Benefits for family life.	Time for family and leisure.	Better social life after work.
	In medicine	40 (13.3)	Family, leisure, quality of life.	Work-hour limitations allow physicians to better recover from work.	My wife is a physician. A semi-normal family life is simply impossible without work-hour limitations.
Efficacy	In general	100 (14.0)	Improved performance.	Efficiency enhancement.	Improved concentration.
	In medicine	24 (8.0)	Enhanced efficacy.	Compulsory introduction of an improvement of organisation and enhanced efficacy.	Maintenance of concentration during work.
Satisfaction, pleasure	In general	61 (8.5)	Greater satisfaction in private life.	Job satisfaction.	Better quality of life.
	In medicine	15 (5.0)	Increase in motivation in a structured work environment.	Happy people work better.	Increment of available apprenticeship places.
Not codeable	In general	7 (1.0)	Beginning of an approach to the work time of officials.	There are only advantages in the prevention of seriously exceeding work-hour limits.	An open-topped competitive relationship is prevented.
	In medicine	29 (9.7)	Enforcement of adapting the teaching.	The whole issue is particularly important for emergency physicians and surgeons.	If there is no regulation, there are no limits for the demands of the chiefs.
Total	In general	716 (100.0)			
	In medicine	300 (100)			

**Table 3:** Examples of surgeons', lawyers', and pilots' arguments against work-hour limitations in general and in medicine.

Category of arguments		N (%)	Surgeons	Lawyers	Pilots
Lack of flexibility	In general	96 (26.5)	Increased handovers lead to a loss of details.	Difficulties in addressing emergencies.	Less flexibility. Work possibly cannot be terminated.
	In medicine	20 (14.7)	There might be medical emergencies that require flexibility.	Flexibility.	Less flexibility for management because of staff shortages.
Costs	In general	69 (19.1)	Rise in personnel costs.	Cost factor.	Cost of health care system.
	In medicine	15 (11.0)	Higher costs.	Health-care costs.	Rise in health insurance costs.
Education	In general	61 (16.9)	Distinct prolonging of surgical training.	Fewer opportunities to learn and to see clinical cases.	Elongation of the training period and of gaining experience.
	In medicine	13 (9.6)	Diminution of experience.	Physicians need spare time for continuing education.	Less time for education.
Quality of work	In general	51 (14.1)	Lack of continuity.	Poor service.	Each person has individual strain limitations that lead to decreased quality caused by overwork.
	In medicine	30 (22.1)	Professional quality is compromised. A limited routine leads to “technocratic medicine”.	Even if it is not only the quality, the treatment might be impaired.	Patients have different contact persons.
Organisability	In general	43 (11.9)	Need for a good organisation.	Lack of enforceability (in certain professions, the failure to comply with work-hour limitations is accepted common practice).	Certain inflexibility in shift scheduling.
	In medicine	35 (25.7)	Planning services is more complicated.	There are patients in need at any time.	Duty rosters are difficult to prepare, especially with regard to limiting the number of days.
Performance	In general	13 (3.6)	Less performance per employee.	Risk of increased pressure on employees leading to their ignoring the work-hour limitations and claiming the wrong effective work hours.	Work-hour limitations are seen as an “optimum” by employers, although they should be considered a “maximum”.
	In medicine	1 (0.7)	Increased number of physicians working at the same place.	-	-
Salary	In general	12 (3.3)	-	The wage level might be moved downwards.	Bonus payments to management might decrease.
	In medicine	4 (2.9)	An absence of overtime is bad for employees.	-	Finances.
Not codeable	In general	17 (4.7)	There is no upper limit.	The wish for diverse employees.	In case of emergencies, temporary exceptions could be applied.
	In medicine	18 (13.2)	This is no contemporary form.	Splitting yes, limitation no.	Public health, occurrence of a catastrophe.
Total	In general	362 (100.0)			
	In medicine	136 (100.0)			

### Comparison of arguments among surgeons, lawyers, and pilots

A comparison among surgeons, lawyers, and pilots regarding the number of arguments in the different categories in favour of and against work-hour limitations is shown in Tables 4 and 5. For all occupation groups combined, there was no difference in the number of arguments for or against work-hour limitations in different categories in terms of relationship status (living with a partner vs. no partner) or hierarchical status (low vs. medium vs. high). Regarding employment level (full-time vs. part-time), age (≤46 vs. >46 years) and work experience, there was no difference for the arguments in favour of work-time regulations. Concerning the arguments against work-time regulations, respondents with full-time jobs cited the argument “quality of work” more often (46/277, 16.6% vs. 5/82, 6.1%; p = 0.017) and cited “costs” less often (45/277, 16.2% vs. 23/82, 28.0%; p = 0.017); participants over 46 years of age cited the argument “education” more often (39/173, 22.5% vs. 20/182, 11.0%; p = 0.003) and cited “lack of flexibility” less often (37/173, 21.4% vs. 58/182, 31.9%; p = 0.026); and the respondents with high work experience cited the arguments “education” (p = 0.044) and “performance” more often (p = 0.003) and cited “costs” less often (0/33, 0% vs. 11/37, 29.7%; p = 0.026). Gender differences in the arguments in favour of and against work-time regulations are depicted in Tables 6 and 7.

**Table 4:** Number of arguments for (n = 716) work-hour limitations given by 65 surgeons, 90 lawyers, and 120 pilots (overall 4 missing values).

Category	Surgeons, N (%)	Lawyers, N (%)	Pilots, N (%)	*P*
Quality, patient safety	19 (13.0)	57 (24.6)	108 (32.0)	**<0.001**
Health, fitness	23 (15.8)	67 (28.9)	93 (27.5)	**0.009**
Leisure, work-family balance	68 (46.6)	51 (22.0)	62 (18.3)	**<0.001**
Efficacy	14 (9.6)	38 (16.4)	48 (14.2)	0.177
Satisfaction, pleasure	17 (11.6)	18 (7.8)	26 (7.7)	0.317
Not codeable	5 (3.4)	1 (0.4)	1 (0.3)	**0.003**
Total	146 (100)	232 (100)	338 (100)	

**Table 5:** Number of arguments against (n = 362) work-hour limitations given by 64 surgeons, 65 lawyers, and 92 pilots (overall 58 missing values).

Category	Surgeons, N (%)	Lawyers, N (%)	Pilots, N (%)	*P*
Lack of flexibility	7 (5.6)	31 (33.7)	58 (40.3)	**<0.001**
Costs	6 (4.8)	20 (21.7)	43 (29.9)	**<0.001**
Education	53 (42.1)	3 (3.3)	5 (3.5)	**<0.001**
Quality of work	36 (28.6)	10 (10.9)	5 (3.5)	**<0.001**
Organisability	16 (12.7)	14 (15.2)	13 (9.0)	0.336
Performance	5 (4.0)	3 (3.3)	5 (3.5)	0.958
Salary	0 (0)	6 (6.5)	6 (4.2)	**0.002**
Not codeable	3 (2.4)	5 (5.4)	9 (6.3)	0.301
Total	126 (100)	92 (100)	144 (100)	

**Table 6:** Number of arguments for (n = 714) work-hour limitations given by 224 male and 48 female surgeons, lawyers, and pilots (overall 7 missing values).

Category	Males, N (%)	Females, N (%)	*P*
Quality, patient safety	160 (27.5)	24 (18.0)	**0.024**
Health, fitness	151 (26.0)	32 (24.1)	0.645
Leisure, work-family balance	143 (24.6)	36 (27.1)	0.556
Efficacy	72 (12.4)	28 (21.1)	**0.009**
Satisfaction, pleasure	48 (8.3)	13 (9.8)	0.575
Not codeable	7 (1.2)	0 (0)	0.203
Total	581 (100)	133 (100)	

**Table 7:** Number of arguments against (n = 362) work-hour limitations given by 181 male and 38 female surgeons, lawyers and pilots (overall 60 missing values).

Category	Males, N (%)	Females, N (%)	*P*
Lack of flexibility	81 (26.6)	15 (25.9)	0.902
Costs	59 (19.4)	10 (17.2)	0.700
Education	55 (18.1)	6 (10.3)	0.149
Quality of work	38 (12.5)	13 (22.4)	**0.047**
Organisability	37 (12.2)	6 (10.3)	0.694
Performance	10 (3.3)	3 (5.2)	0.480
Salary	10 (3.3)	2 (3.4)	0.951
Not codeable	14 (4.6)	3 (5.2)	0.852
Total	304 (100)	58 (100)	

Comparing the participants who agreed with work-time regulations with the remainder, no difference was found in the number of arguments in favour of or against work-time regulations, respectively.

## Discussion

The results of the present report show that the agreement with work-hour limitations in general and in medicine is present and higher for lawyers and pilots than that in surgeons. The latter agreed more with work-hour limitations in general than in medicine. The most often named arguments in favor of work-hour limitations were “quality and patient safety”, “health and fitness”, and “leisure and work-family balance”, whereas the “lack of flexibility” was the most important argument against.

Surgeons, lawyers, and pilots agreed with work-hour limitations in general and medicine. The agreement was higher for lawyers and pilots. It is evident that pilots support work-hour limitations, as they have been accustomed to them for decades to prevent fatigue and to maintain adequate levels of alertness at all times to prevent adverse events [7]. In contrast to lawyers and pilots, surgeons did agree more with work-hour limitations in general than in the medical profession. This corresponds with the publication of Fischer, describing the general surgeon as the last “compleat physician”, who should take care for his patient 24 hours a day, seven days a week [22]. According to Fischer, an 80-hour work-week would be too short a time for surgery residents to provide excellent care [22]. However, this finding also shows that lawyers and pilots did not judge work-hour limitations differently in medicine than in any other work discipline.

Life-style and career orientation have changed: Many physicians are no longer willing to pay a high price for their career at the expense of their lifestyle [23]–[25]. Similarly, we found as principal argument for work-hour limitations by surgeons “leisure and work-family balance”, independent of the marital status. The general opinion among the population is that sleep deprivation results in reduced alertness and jeopardized safety [26],[27]. This is supported by the view of lawyers and pilots as specialists from a legal point of view and experienced executor of work-hour limitations. Furthermore, they named “health and fitness” as main argument for work-hour limitations. It is known that work-hour limitations lead to more time for resting, family, and responsibilities apart from work [28]. In contrast to the view of lawyers and pilots regarding the improved “quality and patient safety”, several studies showed no evidence of reduced morbidity and mortality by reduced work hours [16],[18]. Benefits of a decrease in residents' fatigue might have been reduced due to additional changes of the treating physician [17],[29].

Traditionally, surgical skills are acquired during extended work shifts by observing and by assisting [26]. Not surprisingly, surgeons therefore mainly name their personal “education” and “quality of work” as arguments against work-hour limitations. A previous survey among Swiss surgical residents and attending physicians similarly showed a subjectively clear negative effect on surgical training and on patient care [10]. The reason behind the latter are concerns about loss of continuity in patient care [11],[22],[30].

Lawyers and pilots throw light on the issue from an external point of view, also as a patient, who would like a “flexible” surgeon, who is taking the time for the patient whenever necessary. The lawyers' and pilots' concerns about excessive “costs” incurred due to work-hour limitations is supported by a study of Payette et al., showing that the application of aviation duty-hour restrictions to the health care system would be prohibitively costly [7].

Similar to a survey among senior residents, participants over 46 years and with high work experience, respectively, saw more often an impaired “education” due to work-hour regulations [31]. In contrast to this estimation stand two US studies which showed that a change in work hours is not unconditionally linked with reduced operative caseloads, but may lead to increased operative experience by higher case volumes of senior residents [32],[33].

One of the limitations of this study is its design as an observational study, as it cannot be used to determine a causal relationship between variables. No statement can be made regarding the surgeons, lawyers, and pilots who did not participate in the study and a non-responder bias cannot be excluded. However, compared to other studies in the surgical field, our response rate was high with 56% [34]. To limit the high number of answers resulting from the qualitative pattern of the present study, a random sample of 15% of board-certified surgeons, lawyers and pilots was selected. As a bias regarding the demography cannot be excluded, a comparison of arguments for participants of different age and gender has been performed.

The main strength of this study is that it was conducted with a random sample of surgeons in a variety of working arrangements, covering all of the surgical specializations in the Swiss Surgical Society, of lawyers with the practice area “Labor Law”, and of pilots from all cultural regions in Switzerland.

To our knowledge, this is the first study exploring work-hour limitations in medicine from various professional groups in and outside of surgery: The affected employees, the experts in Labor Law, and persons experienced in dealing with duty-hour restrictions. According to lawyers and pilots, work-hour limitations should lead to better rested physicians with enhanced “quality and patient safety” and improved “health and fitness”. Compared to surgeons, they agreed more often with work-hour limitations, mainly because surgeons expect a threat to their “education” and the “quality of their work”. Surgeons in general agree with work-hour limitations, as they mainly see concurrently an improvement in “leisure and work-family balance”, but they agree less in terms of work-hour limitations in medicine.

It is hard to find rational reasons for lawyers and pilots finding that work-hour limitations in particular should not take place in medicine due to the improvements in safety and well-being shown for other professions. However, to cope with the different opinions, work-hour limitations should be supported, also in medicine, but a way must be found to reduce problems resulting from a discontinuity in patient care and to minimize the work in medicine, which has no education value, without generating excessive “costs”.
